# The *Cryptococcus* wall: A different wall for a unique lifestyle

**DOI:** 10.1371/journal.ppat.1011141

**Published:** 2023-02-23

**Authors:** Liliane Mukaremera

**Affiliations:** Medical Research Council Centre for Medical Mycology at The University of Exeter, University of Exeter, Exeter, United Kingdom; University of Maryland, Baltimore, UNITED STATES

## Introduction

The life-threatening fungal pathogens *Cryptococcus neoformans* and *Cryptococcus gattii* are differentiated from all other human fungal pathogens by the presence of a gelatinous capsule as well as an unusual underlying cell wall. These organisms have both been listed on the WHO list of fungal priority pathogens with higher disease burdens and unmet research and development needs, with *C*. *neoformans* at the top of the critical fungal priority group [1]. The aim of this review is to assess new insights into the unique attributes about *Cryptococcus* cell wall in relation to the pathogenic lifestyle of these important pathogens.

*Cryptococcus* isolates have been classified into two species: *C*. *neoformans* (serotypes A, D, and AD) and *C*. *gattii* (serotypes B and C). Most studies on the *Cryptococcus* cell wall were performed using *C*. *neoformans* strains and, in many cases, without specifying the serotype used. For simplicity, here, I will only use the term “*Cryptococcus*” to indicate *C*. *neoformans* and *C*. *gattii*.

Both the capsule and the cell wall contribute to the virulence properties of *Cryptococcus* and its ability to evade immune detection and killing (Table 1). The capsule and the cell wall are composed of different polysaccharides. While the capsule is mainly composed of glucuronoxylomannan and galactoxylomannan, the cell wall components include alpha- and beta-glucans, chitin, chitosan, and mannoproteins (Table 1). The capsule is an essential virulence factor of *Cryptococcus*, and, as a consequence, its synthesis and function have been extensively studied (reviewed in [2,3]). In contrast, little attention has been paid to the *Cryptococcus* cell wall and its role in pathogenesis. Yet, the cell wall plays a crucial role in capsule synthesis and organisation [4,5], and defects in the *Cryptococcus* cell wall result into dramatic defects in cell division and morphology, increased sensitivity to stresses, and reduced virulence [6–9]. These observations strongly indicate that the cell wall also plays an important role in the biology of *Cryptococcus* and is a driver of *Cryptococcus* infection and disease.

**Table 1:** Chemical signature and function of the *Cryptococcus* cell wall and capsule.

	Components	Function in virulence	Gene involved in the synthesis and/or regulation	Reference
**Cell wall**	Chitin	Increased chitin is associated with nonprotective Th2 immune responses and worsening of the disease	*CHS1*, *CHS2*, *CHS3*, *CHS4*, *CHS5*, *CHS7*, *CHS8*, *CSR1*, *CSR2*, *CSR3*	[3,17]
	Chitosan	Role in maintaining cell wall integrity, bud separation, persistence, and virulence in mammalian hosts.	*CDA1*, *CDA2*, *CDA3*	[6,8,18]
	Chitooligomers or chitin-derived oligomers^1^	Role in the capsule organisation and attachment to the cell wall.	?^2^	[5,19]
	β-1,3-glucans	Essential for cell viability	*FKS1*	[20]
	β-1,6-glucans^3^	Maintains cell wall integrity, cell morphology, and virulence in a mouse model of infection	*KRE5*, *KRE6*, *SKN1*^4^ *KRE61*, *KRE62*, *KRE63*, *KRE64*^5^	[9]
	α-1,3-glucans	Mediates the anchoring of the capsule to the cell wall, cell wall integrity, and virulence	*AGS1*	[4,21]
	Cell wall proteins: The most studied include mannoprotein (MP) 98, 88, 84, 115, and Phospholipase B (Plb1)	MP98 stimulates T cell responses and has properties of a chitin deacetylase.	*CDA2*	[8,22,23]
		MP88 stimulates T cell responses.	*MP88*	
		MP84 and MP115 are putative polysaccharide deacetylase and carboxylesterases, respectively.	*MP84*, *MP115*	
		Plb1 has a role in maintaining cell wall integrity, capsule enlargement, and titan cell formation	*PLB1*	
	Melanin	Protection against environmental stressors, mammalian host defences, and antifungal drugs. Role in virulence in the mouse model of infection.	*LAC1*, *LAC2*	[24,25]
**Capsule**	Glucuronoxylomannan (GXM), 90%–95% of the capsule	Protects against phagocytosis by host phagocytes. Mediates survival inside phagolysosomes, and dissemination to the brain. Secreted capsule polysaccharides induce immunological unresponsiveness. GXM and GalXM have immunomodulatory properties—anti-inflammatory in macrophages, but pro-inflammatory in neutrophils	*CAP59*, *CAP60*, *CAP64*, *CAP10*, *UGE1*, *UGM*, *UGT1*, *MAN1*, *UGD1*, *UXS1*, *GMT1*, *GMT2*, *UUT1*, *UXT1*, *UXT2*, *CAS1*, *CXT1*	[2,3]
	Glucuronoxylomannogalactan (GXMGal), also known as Galactoxylomannan (GalXM), 5%–8% of capsule			
	Mannoproteins (<1%)^6^	?	?	

^1^Structures with properties similar to chitin (composed of N-acetyl glucosamine and sensitives to chitinases), which bind the Wheat Germ Agglutinin, but not Calcofluor White (a fluorescent stain that binds to chitin).

^2^Thought to be derived from chitin, but synthesis not fully understood.

^3^*KRE* genes (*KRE5*, *KRE6*, *SKN1*) are involved in the β-1,6-glucan synthesis, but the exact mechanisms are not fully understood.

^4^*kre5*Δ mutant and *kre6*Δ*/skn1*Δ double mutants have less β-1,6-glucans, are sensitive to cell wall inhibitors, and were avirulent in mice.

^5^*Cryptococcus* genes that are homologs to *Saccharomyces cerevisiae KRE6* gene. Disruption of *KRE6* gene results into the reduction of β-1,6-glucan levels in *S*. *cerevisiae*, but the phenotypes of *Cryptococcus* mutants are similar to the wild-type strain. The virulence in mice was not tested for *Cryptococcus* mutants.

^6^Function and role in the capsule architecture are currently not well understood.

## *Cryptococcus* cell wall composition differs from that of other major human fungal pathogens

The *Cryptococcus* cell wall is mainly composed of glucans (α-1,3-glucan, β-1,3-glucan, and β-1,6-glucan), glycoproteins, chitin, and its deacelylated form chitosan (Table 1 and reviewed in [3]), but also contains melanin and lipids [10,11]. Although most fungal cell walls consist of similar polysaccharides, differences between *Cryptococcus* cell wall and the walls of other common fungal pathogens have been observed.

### β-1,3-glucans and β-1,6-glucans

Unlike *Candida albicans* or *Saccharomyces cerevisiae*, the *Cryptococcus* cell wall contains more β-1,6-glucan than β-1,3-glucan [3,12]. *Cryptococcus* β-1,6-glucan is involved in cell wall organisation through its interaction with chitin, β-1,3-glucan, and glycoproteins [3]. In addition, mutants with defects in β-1,6-glucans form diffuse and enlarged capsules with rough edges, contrary to the smooth edges of the wild-type strains [9]. Although *Cryptococcus* contains reduced amounts of β-1,3-glucan, they are nonetheless important. The one gene *FKS1* encoding for β-1,3-glucan in *Cryptococcus* is essential. Therefore, β-glucans are vital for *Cryptococcus* viability and capsule organisation.

### Chitosan

Compared to other major human fungal pathogens, *Cryptococcus* cells wall contains relatively high amounts of chitosan. In *Cryptococcus*, the wall chitosan content is 3 to 5 times higher than chitin during vegetative growth [13]. This is similar to the less clinically common zygomycete pathogens where 65% to 95% of the chitin is deacetylated [14]. Chitosan is also present in the ascospores of *S*. *cerevisiae* and the chlamydospores of *Candida dubliniensis* in small amounts, but absent in the vegetative cell wall of the yeast cells [15,16]. The cell wall of major pathogens *Aspergillus fumigatus*, *C*. *albicans*, and *Pneumocystis jirovecii* contain little or immeasurable chitosan. In *Cryptococcus*, chitosan is present in both *in vitro*-grown cells and cells isolated from infected mice [6,17], and chitosan deficiency has been associated with a reduced virulence [6]. Therefore, the *Cryptococcus* cell wall is particular in containing chitosan in both vegetative growth and *in vivo*, and chitosan is required for *Cryptococcus* pathogenesis.

## The cell wall structure varies between *Cryptococcus* yeast cells of different sizes

The fungal cell wall is a dynamic and flexible structure that change significantly in composition during normal cell growth, environmental adaptation, or during morphological transitions. When grown in standard laboratory growth conditions, *Cryptococcus* cells appear as a homogenous population of 5 to 7 μm “normal-sized” yeasts [26]. In contrast, yeast cells extracted from infected tissues are of varying sizes and morphological characteristics [26,27]. This dynamic population includes greatly enlarged cells called “titan cells” (10 to 100 μm in diameter), “normal-sized” yeasts, and smaller cells (less than 4 μm of diameter) called titanides, seeder cells, and micro/drop cells [26–29]. Titan cells are so large that they may present challenges to efficient immune cell phagocytosis [26]. In addition to differences in cell sizes, these cell populations present differences in the structure of their cell wall. Titan cells have a significantly thicker cell wall (2 to 3 μm) than normal-sized cells (0.05 to 0.1 μm) [30] and have increased chitin and mannose contents [17,31]. Titanides, seeder, and micro/drop cells are small, and their cell wall structure also differs significantly from normal yeasts. Drop cells are round and have a thicker cell wall [28], while titanides are oval and have a thin cell wall [29]. The newly characterised seeder cell population is similar in size to titanides and have more exposed mannan than larger cells [27]. Therefore, the host immune system must be capable of recognising *Cryptococcus* yeast cells with significant differences in their size as well as their capsule and cell wall composition. However, the precise role of each morphotype in the immune recognition of *Cryptococcus* is not fully understood.

## Both the capsule and the cell wall of *Cryptococcus* wall influence the host immune response

Because it is enveloped by the capsule, it is not clear how the cell wall actively engages in immune activation. However, it is clear that the wall also contributes to immune recognition and the immune response to this fungus (Table 1; [17,32]). Chitin and chitosan have been associated with nonprotective immune responses [17,18], although they are in the inner layer of the cell wall and covered by other wall components and by the capsule. This is problematic in understanding how the interaction between chitin/chitosan and immune cells occurs, or whether it is triggered by intact cells or by cell wall fragments that are shed by the yeast cell. Cell wall β-1,3-glucans have been detected in the cerebrospinal fluid and serum of HIV+ patients with *Cryptococcus* meningitis and were associated with pro-inflammatory chemokine responses [33]. In addition, mannoproteins recovered from the *Cryptococcus* culture supernatant stimulate T-cell immune responses [22,23]. *Cryptococcus* releases capsule polysaccharides into the extracellular space during infection and in *in vitro* culture, and the shed polysaccharides modulate the host immune responses [32]. Similarly, cell wall components may be shed and interact with the host cells indirectly. It is not known whether cell wall components, other than β-1,3-glucans and mannoproteins, are also shed during *Cryptococcus* infection and contribute to immune stimulation.

## The cell wall and limitations in the use of antifungal drugs

Echinocandin antifungal drugs (caspofungin, anidulafungin, and micafungin) inhibit β-1,3-glucan synthesis, resulting in the disruption of cell wall integrity and, ultimately, fungal cell death [34]. Although echinocandins are active against most *Candida* and *Aspergillus* species, they are largely ineffective against *Cryptococcus* *in vivo* [34,35]. This is surprising in so far as the *FKS1* gene that encodes for β-1,3-glucan synthase is essential in *Cryptococcus* [20], and this enzyme is sensitive to echinocandins *in vitro* [36]. The mechanisms behind the resistance to echinocandins are not well understood.

In comparison to other yeasts, *Cryptococcus* cell wall contains more β-1,6-glucans than β-1,3-glucans. Could this difference in β-glucans impact the resistance of *Cryptococcus* to echinocandins? A previous study showed that treating *Cryptococcus* with caspofungin resulted in the reduction of both β-1,3-glucans and β-1,6-glucans, and concluded that inhibition of β-1,6-glucans may be an additional mechanism of action of pneumocandin [37]. Therefore, increased β-1,6-glucans in *Cryptococcus* cell wall does not explain its resistance to echinocandins.

Another possibility is that *in vivo* cell adaptations such as the capsule and the thick cell wall could prevent access of echinocandins to their target enzyme. Studies using acapsular and melanin-deficient mutants found that the capsule and melanin were not required for the caspofungin resistance [38]. However, lipid flippase defects in the cell membrane were associated with higher caspofungin penetration into the cell and increased caspofungin susceptibility [38]. In response to caspofungin, *Cryptococcus* increased its chitin and chitosan contents [39], a compensatory mechanism similar that observed in *Candida* species and *A*. *fumigatus* [40]. Therefore, both the cell wall and plasma membrane integrity may play a role in *Cryptococcus* resistance to echinocandins.

Chitin synthase inhibitors have been investigated as antifungal drugs and some (e.g. Nikkomycins) have shown *in vitro* and *in vivo* activity against fungal pathogens such as *Coccidioides* and *Blastomyces* species [41]. These chitin synthase inhibitors do not have a strong activity against *Cryptococcus*, and currently, there is no chitin synthase inhibitor in clinical use.

## Concluding remarks

The fungal wall is an ideal target for the development of new antifungal drugs. *Cryptococcus* cell wall differs in design and composition from that of other major human fungal pathogens. Although substantial work is still needed to fully understand the role of each wall component in immune recognition/evasion and/or antifungal drug resistance, information presented here emphasizes that the cell wall is a key player in *Cryptococcus* pathogenicity and could be a potential target of new anti-*Cryptococcus* drugs.
